# Supplementary material for: Structural and mechanistic insights into activation of the human RNA ligase RTCB by Archease
Source: Nat Commun. 2024 Mar 16;15:2378. doi: 10.1038/s41467-024-46568-2 (PMC10944509; doi:10.1038/s41467-024-46568-2)
Supplement: Supplementary file 1 — Supplementary Information [file 41467_2024_46568_MOESM1_ESM.pdf]

Supplementary Information for:

Structural and mechanistic insights into activation of the human RNA ligase RTCB by Archease

Janina Lara Gerber[1], Suria Itzel Morales Guzmán[1], Lorenz Worf[1], Petra Hubbe[1], Jürgen Kopp[1] and Jirka Peschek[1]*

[1] Heidelberg University, Biochemistry Center (BZH), Im Neuenheimer Feld 328, 69120 Heidelberg, Germany

* Corresponding author: Jirka Peschek, jirka.peschek@bzh.uni-heidelberg.de

Supplementary Fig. 1 Recombinant human RTCB and Archease.

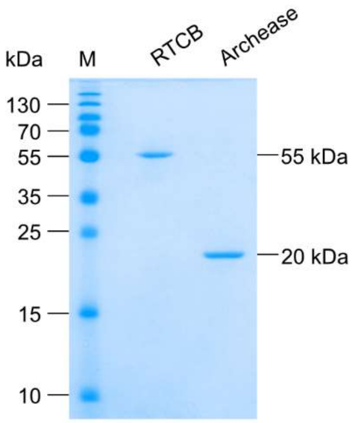

Purified proteins were analyzed by SDS-PAGE and stained with Coomassie blue. Source data are provided as a Source Data file.

Supplementary Fig. 2 Effect of Archease and nucleotide on RTCB-catalyzed RNA ligation.

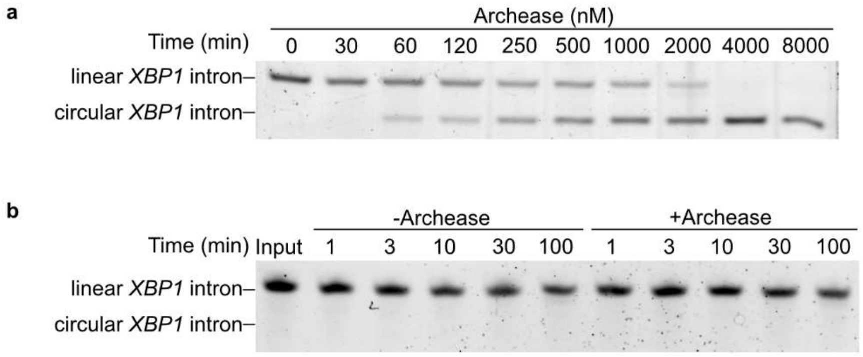

a Concentration-dependent ligation of *XBP1* intron by RTCB (1 µM) in the presence of the cofactors $MnCl_2$ (0.5 mM) and GTP (1 mM) and indicated concentrations of Archease were incubated at 30°C for 30 min. Aliquots were taken and analyzed by denaturing urea-PAGE. **b** In vitro ligation of the *XBP1* intron by RTCB (250 nM) in the absence or presence of Archease (1 µM). Ligation reactions were performed with ATP (1 mM) as nucleotide co-factor. Reactions were performed at 30°C. Aliquots were taken and quenched at different time points and analyzed by denaturing urea-PAGE.
Source data are provided as a Source Data file.

Supplementary Fig. 3 Sequence and structural alignment of Archease.

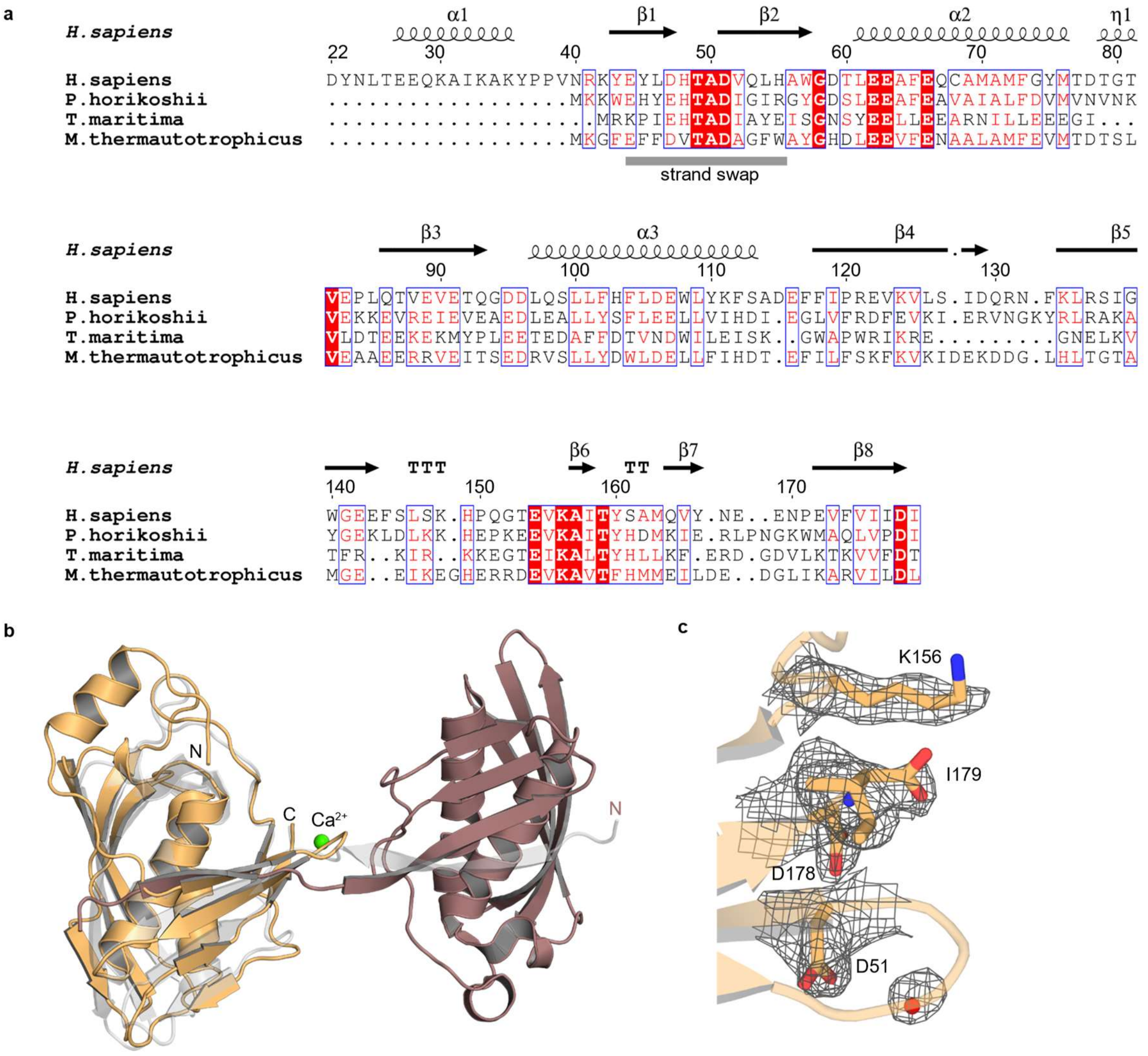

a Sequence alignment of Archease and three homologous enzymes from *P. horikoshii, T. maritima* and *M. thermautotrophicus* respectively (created with ESPript 3.0). Secondary structure elements are indicated based on the human Archease structure as follows: α-helices (α), 3_{10}-helix (η), α-turn (TTT) and β-turn (TT). Residues with red background and white characters indicate strictly conserved residues, red characters indicate residues with high similarity (Similarity Global Score > 0.7), both are grouped together by blue frames.

b Superposition of human Archease (orange) with *P. horikoshii* Archease (PDB ID: 4N2P; brown and grey) represented as cartoons. The Ca^{2+} ion from the *Ph*Archease structure is shown as a green sphere.

c Detailed view of conserved residues of Archease (orange). Waters (red) are depicted as spheres. The $2mF_o$-DF_c simulated annealing omit map is shown around the indicated residues (grey mesh), contoured at 1 σ.

Supplementary Fig. 4 Comparison of the RTCB-Archease complex with previous crystal structures.

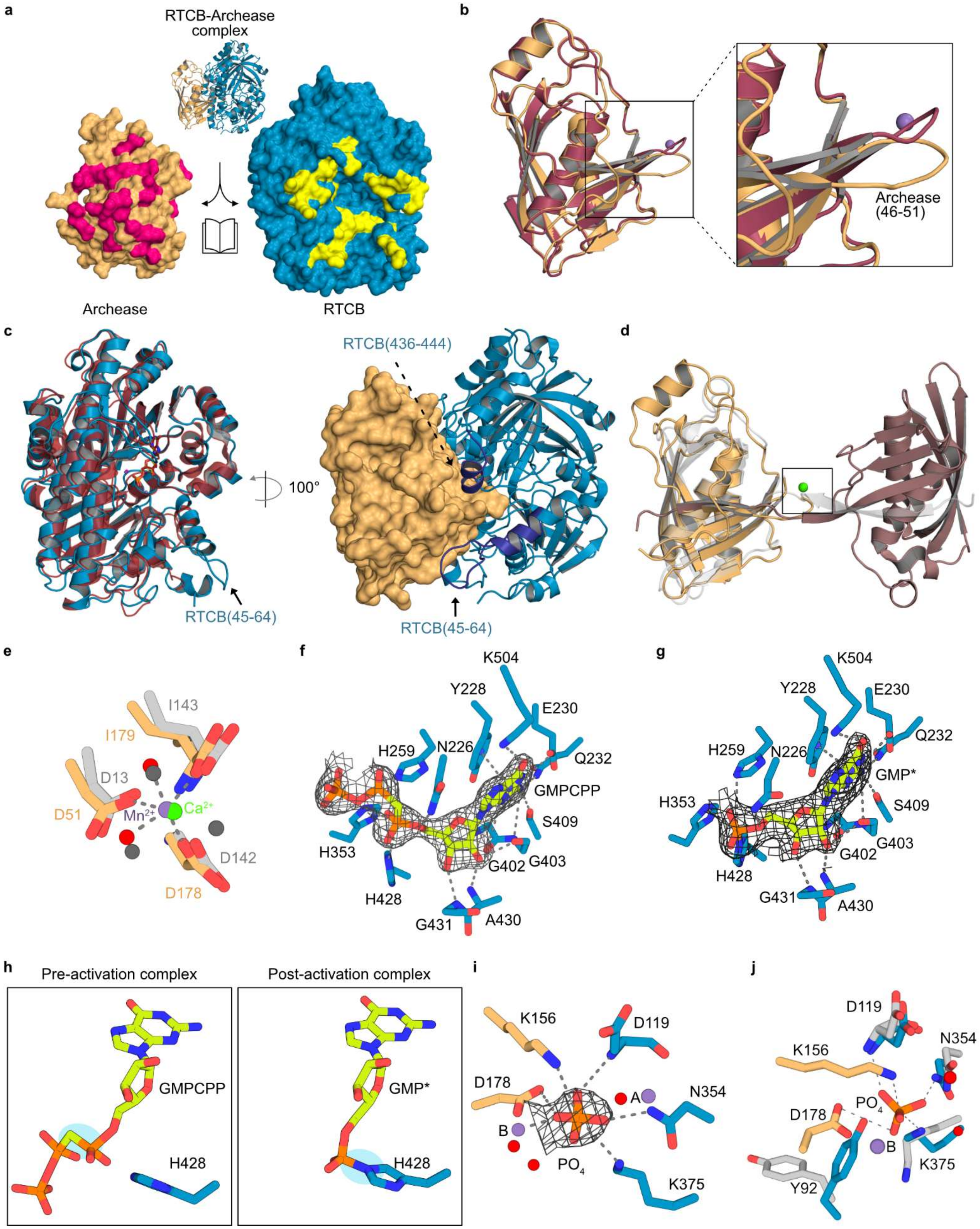

a RTCB (teal) and Archease (orange) complex depicted as surface representation. The interaction surface is highlighted in yellow for RTCB and in pink for Archease. Interacting residues are listed in Supplementary Data 1. **b** Left: Superposition of monomeric Archease (red) and Archease from the RTCB-Archease complex with GMP (orange). The Mn^{2+}(C) ion is depicted as purple sphere in all panels. **c** Left: Superposition of RTCB, from the RTCB-Archease complex with GMP, in teal and *Ph*RtcB (PDB ID: 4DWQ) in red. Right: RTCB (teal) and Archease (orange) in complex with GMP. Regions 45-64 and 436-444 are highlighted in dark blue. Archease is depicted as surface and RTCB in cartoon representation. **d** Superposition of the Archease structure from the pre-activation complex and the *P. horikoshii* dimer structure of Archease (PDB ID: 4N2P) with Ca^{2+} (green) in brown and grey. **e** Superposition of metal binding residues of human Archease (orange) and *P. horikoshii* Archease (grey). Depicted is the boxed region of panel **d**. Mn^{2+} ions (purple) and waters (red) of human Archease are depicted as spheres. Ca^{2+} is shown as light grey sphere and water of *Ph*Archease are shown as dark grey spheres. The hydrogen bonds and metal coordination bonds of the human Archease are shown as grey dashed lines. **f** Detailed view of GMPCPP interacting RTCB residues. The GMPCPP is shown as sticks (lime), Mn^{2+} is shown as purple sphere. The hydrogen bonds and metal coordination bonds are shown as grey dashed lines. The $2mF_o$-DF_c simulated annealing omit map is shown around GMPCPP (grey mesh), contoured at 1 σ. **g** Detailed view of GMP* interacting RTCB residues. The guanylylated GMP-His428 is marked as GMP* and shown as sticks (lime), Mn^{2+} is shown as purple sphere. The hydrogen bonds and metal coordination bonds are shown as grey dashed lines. The $2mF_o$-DF_c simulated annealing omit map is shown around GMP* (grey mesh), contoured at 1 σ. **h** Human RTCB (teal) active site in different activation states in presence of Archease (orange). GMP* and GMPCPP are shown as sticks (lime). The covalent at the α-phosphate, which is inverted during the phosphoryl transfer reaction, is highlighted (light blue background). Left: Pre-activation RTCB active site with GMPCPP as nucleotide co-factor. Right: Post-activation RTCB active site. The guanylylated GMP-His428 is marked as GMP*. **i** Detailed view of PO_4-interacting residues of RTCB (teal) and Archease (orange). PO_4 is depicted as orange sticks. Mn^{2+} ions (purple) and waters (red) are depicted as spheres. The hydrogen bonds and salt bridges are shown as grey dashed lines. The $2mF_o$-DF_c simulated annealing omit map is shown around the phosphate (grey mesh), contoured at 1 σ. **j** Superposition of PO_4-interacting residues of RTCB (teal) from the post-activation complex with Archease (orange) and nucleotide free RTCB (grey). PO_4 is depicted as orange sticks. Mn^{2+} ions (purple) are depicted as spheres. The hydrogen bonds and salt bridges are shown as grey dashed lines.

Supplementary Fig. 5 Omit map of nucleotide-free RTCB.

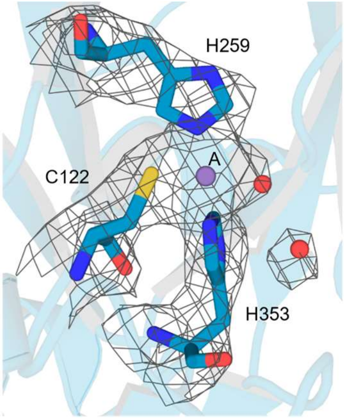

Detailed view of Mn^{2+}(A)-interacting residues of nucleotide-free RTCB (teal). Mn^{2+} ions (purple) and waters (red) are depicted as spheres. The $2mF_o$-DF_c simulated annealing omit map is shown around the indicated Mn^{2+}(A)-interacting residues (grey mesh), contoured at 1.5 σ.

Supplementary References

1. Krissinel, E. & Henrick, K. Inference of Macromolecular Assemblies from Crystalline State. *Journal of Molecular Biology* **372**, 774–797 (2007).
