# Supplementary material for: Structural and mechanistic insights into activation of the human RNA ligase RTCB by Archease
Source: Nat Commun. 2024 Mar 16;15:2378. doi: 10.1038/s41467-024-46568-2 (PMC10944509; doi:10.1038/s41467-024-46568-2)
Supplement: Supplementary file 3 — Description of Additional Supplementary Files [file 41467_2024_46568_MOESM3_ESM.pdf]

Description of Additional Supplementary Files:

Supplementary Data 1: Interaction interface of RTCB and Archease, calculated by the PISA server.

Supplementary Movie 1: The conformational changes between nucleotide-free RTCB and RTCB in the activation complex are visualized as morph (generated in PyMol). The loop region Q218 to Y228 is highlighted in red. Loops that were not build for the nucleotide free RTCB were deleted in the RTCB structure from the post-activation complex for comparison.
